# The Effect of Intravenous Vitamin C on Cancer- and Chemotherapy-Related Fatigue and Quality of Life

**DOI:** 10.3389/fonc.2014.00283

**Published:** 2014-10-16

**Authors:** Anitra C. Carr, Margreet C. M. Vissers, John S. Cook

**Affiliations:** ^1^Department of Pathology, Centre for Free Radical Research, University of Otago, Christchurch, New Zealand; ^2^New Brighton Health Care, Christchurch, New Zealand

**Keywords:** vitamin C, cancer, chemotherapy, fatigue, quality of life

## Abstract

Cancer patients commonly experience a number of symptoms of disease progression and the side-effects of radiation therapy and adjuvant chemotherapy, which adversely impact on their quality of life (QOL). Fatigue is one of the most common and debilitating symptom reported by cancer patients and can affect QOL more than pain. Several recent studies have indicated that intravenous (IV) vitamin C alleviates a number of cancer- and chemotherapy-related symptoms, such as fatigue, insomnia, loss of appetite, nausea, and pain. Improvements in physical, role, cognitive, emotional, and social functioning, as well as an improvement in overall health, were also observed. In this mini review, we briefly cover the methods commonly used to assess health-related QOL in cancer patients, and describe the few recent studies examining the effects of IV vitamin C on cancer- and chemotherapy-related QOL. We discuss potential mechanisms that might explain an improvement in QOL and also considerations for future studies.

## Introduction

High-dose intravenous (IV) vitamin C has been administered by physicians for many decades as a complementary and alternative therapy for cancer patients (1). Although the use of IV vitamin C is relatively common, there is still controversy as to its efficacy in the treatment of cancer (2). Much of the controversy can be attributed to the lack of an agreed mechanism of action, and numerous issues around study design, including a lack of understanding of vitamin C pharmacokinetics (3). Vitamin C administered intravenously bypasses the regulated intestinal uptake mechanism and results in significantly higher plasma concentrations than are obtained through oral intake (4). It is proposed that at these high-doses vitamin C acts as a pro-drug via metal ion-dependent generation of cytotoxic hydrogen peroxide (3), however, other potential anti-cancer mechanisms, such as regulation of epigenetic marks and transcription factors, are also possible (5).

Recently, evidence has been accumulating which indicates that IV vitamin C may improve the quality of life (QOL) of cancer patients, both in the presence and absence of adjuvant chemo- and radiotherapy (6–9). Two early studies of high-dose vitamin C in patients with advanced cancer indicated that many of the patients experienced some subjective improvement in QOL, including reduced pain and the need for analgesics (10, 11). The authors stated that “any agent which can make, or even appear to make, the burden of terminal cancer more tolerable, deserves further study.” Health-related QOL encompasses the general well-being of an individual and comprises both objective and subjective measures, the latter of which can be difficult to measure and quantify. In this mini review, we briefly cover the methods commonly used to assess health-related QOL in cancer patients, and describe the few recent studies examining the effects of IV vitamin C on cancer- and chemotherapy-related QOL. We discuss potential mechanisms that might explain an improvement in QOL and also considerations for future studies.

## Quality of Life and Fatigue Measures

Numerous instruments exist that can be used to assess health-related QOL in cancer patients (12). These include general measures such as the short form 36 (SF-36) and the EuroQoL 5 dimensions (EQ-5D), as well as specific measures such as the functional assessment of cancer therapy-general (FACT-G) and the European organization for research and treatment of cancer quality of life questionnaire (EORTC QLQ-C30) (13). The UK national institute for health and clinical practice (NICE) recommends generic measures such as the EQ-5D in order to compare different disease conditions, however, the EQ-5D has been criticized for its lack of sensitivity with cancer patients (14), and the SF-36 also does not appear to perform as well as the EORTC QLQ-C30 (15). The FACT-G and EORTC QLQ-C30 perform equally well (13) and three of the four studies assessing the effect of IV vitamin C on cancer-related QOL have used the EORTC QLQ-C30 (6, 8, 9). The EORTC QLQ-C30 is a reliable and valid QOL questionnaire comprising 30 core items (16). Multi-item scales include five functional scales (physical, role, cognitive, emotional, and social), three symptom scales (fatigue, pain, and nausea/vomiting), and a global health and QOL scale. Additional single item scales include symptoms commonly reported by cancer patients (e.g., dyspnea, appetite loss, sleep disturbance, constipation, and diarrhea) as well as the financial impact of the disease and treatment. The measures range in score from 0 to 100, with a difference of 4–10 points representing a small change, and a difference of 10–20 points representing a medium change in QOL (17).

Fatigue is one of the most common and debilitating symptom reported by cancer patients and can affect QOL more than pain (18). Numerous measures of fatigue have been developed, including the fatigue severity scale (FSS), fatigue impact scale (FIS), and the brief fatigue inventory (BFI) (19). However, fatigue is not one-dimensional, i.e., it has physical, emotional, and mental aspects, therefore, multidimensional measures offer more comprehensive information. These measures include the fatigue symptom inventory (FSI), multidimensional assessment of fatigue (MAF), and multidimensional fatigue symptom inventory (MFSI) (19). Some instruments have been validated for the measurement of cancer-related fatigue and are therefore preferred, e.g., BFI and MFSI (20). The use of standardized instruments facilitates cross study comparisons. We have used the MFSI-SF (21) to assess the effects of IV vitamin C on the multidimensional aspects of cancer- and chemotherapy-related fatigue (22, 23).

## Chemotherapy-Related Quality of Life

Patients undergoing chemotherapy commonly experience fatigue, with more than 75% of patients reporting feelings of debilitating tiredness or loss of energy (18). Fatigue has a constant presence following chemotherapy, increases incrementally with consecutive cycles of chemotherapy (24), and may persist for years after treatment completion (25). Although cancer-related pain can be managed with opioids (26), no effective therapy for fatigue has yet been identified. The efficacy of IV vitamin C administration on the QOL of breast cancer patients during chemo-/radiotherapy and aftercare was investigated in a retrospective cohort study (7). A total of 53 patients were treated with 7.5 g vitamin C once a week for a minimum of 4 weeks (not on the days of chemo-/radiotherapy) and compared with 72 controls. Intensity of complaints was measured on a scale of 0 = no complaints, 1 = mild complaints, and 2 = severe complaints. Significantly decreased fatigue, depression, sleep disorders, and loss of appetite were observed in the treatment group compared with the control group during chemo-/radiotherapy (Table 1). During the after-care phase, a significant decrease in fatigue, sleep disorders, dizziness, nausea, and loss of appetite was observed in the treatment group.

**Table 1:** **Intravenous vitamin C and cancer-/chemotherapy-related quality of life**.

Study type	Patients	Intervention	Other therapies	Measures	Outcomes	
**PROSPECTIVE**						
Ma et al. (27)	25 Ovarian cancer (stage III–IV) 13 Chemo + vitC group 12 Chemo group	IV vitamin C 75–100 g 2 × /week 12 months	± Chemotherapy (paclitaxel, carboplatin) 6 months	Adverse events/toxicity	↓ Grade 1 and 2 toxicities (neurological, bone marrow, hepatobiliary/pancreatic, renal/genitourinary, pulmonary, infection, gastrointestinal, and dermatological)	
Stephenson et al. (8)	17 Refractory advanced solid tumors (stage III–IV; colon, pancreas, breast, etc.)	IV vitamin C 0.8–3 g/kg 4 × /week 1–4 weeks	None	EORTC QLQ-C30^a^	↓ Fatigue ↓ Pain ↓ Nausea/vomiting ↓ Insomnia ↓ Appetite loss	↑ Global health ↑ Physical, role, emotional, cognitive, and social functioning
Takahashi et al. (9)	60 Advanced cancer (lung, breast, stomach, colon, etc)	IV vitamin C 25–100 g 2 × /week 4 weeks	± Chemotherapy	EORTC QLQ-C30	↓ Fatigue ↓ Pain ↓ Insomnia ↓ Constipation	↑ Global health ↑ Physical, role, emotional, cognitive, and social functioning
Yeom et al. (6)	39 Terminal cancer (stomach, colorectal, lung, breast, biliary, etc)	IV vitamin C 10 g 2 × /week 4 g oral daily 1 week	None	EORTC QLQ-C30	↓ Fatigue ↓ Pain ↓ Nausea/vomiting ↓ Insomnia ↓ Appetite loss	↑ Global health ↑ Physical, role, emotional, cognitive, and social functioning
**CASE STUDIES**						
Carr et al. (22)	1 Breast cancer	IV vitamin C 50 g 2 × /week 4 weeks	Chemotherapy (doxorubicin, cyclophosphamide, paclitaxel)	EORTC QLQ-C30	↓ Fatigue ↓ Pain ↓ Nausea/vomiting ↓ Insomnia ↓ Appetite loss	↑ Global health ↑ Physical, emotional, cognitive, and social functioning
				MFSI^b^	↓ General, physical, emotional, mental, and total fatigue	↑ Vigor
Carr et al. (23)	1 Terminal angiosarcoma	IV vitamin C 30 g daily 1 week	None	EORTC QLQ-C30	↓ Fatigue ↓ Pain ↓ Nausea/vomiting ↓ Insomnia ↓ Appetite loss	↑ Global health ↑ Physical, emotional, cognitive, and social functioning
				MFSI	↓ General, physical, emotional, mental, and total fatigue	
**RETROSPECTIVE**						
Vollbracht et al. (7)	125 Breast cancer (stage IIa–IIIb) 53 Treatment group 72 Control group	IV vitamin C 7.5 g 1 × /week ≥4 weeks	± Chemotherapy (epirubicin, cyclophosphamide, methotrexate, fluorouracil) ± Radiotherapy	Complaints 0 = no complaint 1 = mild complaint 2 = severe complaint	↓ Fatigue ↓ Depression ↓ Nausea ↓ Sleep disturbance ↓ Appetite loss ↓ Dizziness	

*^a^EORTC QLQ-C30, European organization for the research and treatment of cancer quality of life questionnaire – core 30 (16)*.

*^b^MFSI, multidimensional fatigue symptom inventory (21)*.

A subsequent prospective intervention study investigated the health-related QOL of 60 patients with advanced cancer, about half of whom were undergoing chemotherapy during the study period (9). IV vitamin C (25–100 g/session) was administered twice a week and EORTC QLQ-C30 questionnaires were completed before and following 2 and 4 weeks of vitamin C intervention. A statistically significant decrease in fatigue, insomnia, and constipation was observed following 2 weeks, and a reduction in pain following 4 weeks of intervention (Table 1). Significant improvements in physical, role, emotional, and social function were observed following 2 weeks and an improvement in cognitive functioning following 4 weeks. Overall, the patients’ global health status improved from a score of 45 to a score of 61 after 4 weeks of IV vitamin C.

We carried out a case study of a 45-year-old female diagnosed with breast cancer (22). Lethargy was a major symptom of chemotherapy (doxorubicin, cyclophosphamide, and paclitaxel). To investigate the effects of pharmacologic vitamin C on QOL and fatigue due to chemotherapy, IV vitamin C (50 g/session) was initiated twice weekly, 2 days either side of each chemotherapy session. QOL (EORTC QLQ-C30) and fatigue (MFSI-SF) questionnaires were undertaken before and after 4 weeks of vitamin C intervention (16, 21). The QOL questionnaire showed dramatic decreases in fatigue, pain, appetite loss, nausea/vomiting, and insomnia following vitamin C administration (Table 1). Increases in physical, emotional, cognitive, and social functioning were also observed, as well as a doubling of the patient’s “global health status.” The multidimensional fatigue symptomology questionnaire showed decreases in general, physical, emotional, and mental fatigue, as well as increased vigor, following vitamin C administration.

Although not addressing QOL directly, a recent translational study showed reduced chemotherapy-related toxicity when high-dose IV vitamin C was administered in conjunction with carboplatin and paclitaxel (27). Twenty-five patients with stage III–IV ovarian cancer were randomized to receive either chemotherapy alone (for 6 months) or chemotherapy plus IV vitamin C (75–100 g two times per week for 12 months). Adverse events were evaluated and grade 1 and 2 toxicities were found to be significantly decreased in the group receiving IV vitamin C (Table 1). Decreased toxicities were observed in almost all the evaluated categories, e.g., neurological, bone marrow, hepatobiliary/pancreatic, renal/genitourinary, infection, pulmonary, gastrointestinal, and dermatological. Interestingly, the decreased toxicity did not adversely affect survival of the patients, suggesting that IV vitamin C is a potentially useful adjuvant to some chemotherapeutic agents. In support of a role for vitamin C in decreasing chemotherapy-related toxicity, a phase I clinical study of pharmacologic vitamin C in combination with gemcitabine (for the control of pancreatic cancer) reported decreased plasma F_2_-isoprostanes, a well validated biomarker for endogenous oxidative stress, following administration of IV vitamin C (28).

## Palliative Care of End-Stage Patients

In patients with advanced cancer, where a cure is unlikely, QOL becomes an important and highly valued consideration for both the patients and their families. Yeom et al. (6) carried out a prospective study of 39 terminal cancer patients who were not undergoing adjuvant chemo-/radiotherapy. The patients were administered IV vitamin C (10 g twice over 3 days) followed by oral vitamin C intake (4 g/day for 1 week). The EORTC QLQ-C30 was used to assess QOL before and after vitamin C administration. The patients reported significantly lower scores for fatigue, pain, nausea/vomiting, and appetite loss following administration of vitamin C (Table 1). The patients also reported significantly higher scores for physical, role, emotional, and cognitive function, as well as an overall improvement in their global health scale (from a score of 36 to a score of 55) following vitamin C administration.

A recent phase I clinical trial designed to test the safety, tolerability, and pharmacokinetics of IV vitamin C in patients with advanced cancer also measured health-related QOL using the EORTC QLQ-C30 (8). Patients with refractory advanced solid tumors (*n* = 17) were treated with IV vitamin C at 0.8–3 g/kg body weight for 1–4 weeks. The QOL assessment gave variable data depending on the week and sample size, but overall there appeared to be an improvement in most functioning and symptom scales for weeks 2 and 4 (Table 1).

We carried out a case study of an 81-year-old male with inoperable pulmonary angiosarcoma (23). IV vitamin C (30 g/session) was initiated and QOL (EORTC QLQ-C30) and fatigue (MFSI-SF) questionnaires were administered before and after seven consecutive days of vitamin C administration. The QOL questionnaire showed a 37% decrease in fatigue and complete cessation of pain, nausea/vomiting, and insomnia, as well as ameliorated loss of appetite, following vitamin C administration (Table 1). Improvements in physical, emotional, cognitive, and social functioning were also observed, as well as an enhancement of overall QOL. With respect to the multidimensional aspects of fatigue, a decrease in physical, emotional, and mental fatigue, resulting in a 50% decrease in total fatigue, was observed following vitamin C administration.

## Potential Mechanisms of the Quality of Life Action

A number of studies have reported low-plasma levels of vitamin C in cancer patients and in some instances this can amount to severe depletion (29, 30). However, the vitamin C status of cancer patients is not often taken into consideration, despite its potential to support many biological functions. Vitamin C is well known for its potent antioxidant properties, being able to readily scavenge free radicals and reactive oxygen species, and is associated with decreased markers of oxidative stress *in vivo* (31, 32). Oxidative stress is a major etiological factor in the development and progression of cancer (33), and radiotherapy and some chemotherapeutic agents also act through increased production of free radicals. Oxidative stress is thought to contribute to chemotherapy-related weakness and fatigue (34), thus, vitamin C may be able to decrease fatigue through decreasing off-target oxidant-mediated toxicity of adjuvant therapy (27, 28). There is also strong evidence implicating inflammation in the sequelae of cancer-related fatigue (25) and preliminary studies have shown that high-dose IV vitamin C reduces biomarkers of inflammation, such as C-reactive protein (CRP), tumor necrosis factor (TNF-α), interferon-γ (IFN-γ), and the interleukins IL-1, IL2, IL-6, IL-8, in cancer patients (35, 36).

Vitamin C acts as an *in vivo* cofactor for a family of enzymes involved in the biosynthesis of collagen, carnitine, neurotransmitters, and neuropeptide hormones, as well as those involved in the regulation of epigenetic marks and transcription factors (5). Some of these mechanisms may account for its observed effects on QOL. Vitamin C is a cofactor for two enzymes involved in the biosynthesis of carnitine, which is required for the transport of fatty acids into mitochondria during the breakdown of lipids for the generation of metabolic energy, i.e., ATP (37); this suggests a plausible mechanism by which vitamin C could alleviate physical fatigue. Parenteral vitamin C (1.5–15 g/session) has been shown to readily and rapidly reduce pain in patients with shingles (38, 39) and although the mechanism of its analgesic action is uncertain, it is likely to be through its neuromodulatory functions (40). Vitamin C acts as a cofactor in the synthesis of the neurotransmitters norepinephrine, dopamine, and serotonin (the latter two via recycling the enzyme cofactor tetrahydrobiopterin) (41), and in the synthesis of neuropeptide hormones, such as oxytocin (42), which has known mood enhancing effects. To date, no cancer- or chemotherapy-related QOL studies have investigated the effect of IV vitamin C on biomarkers of oxidative stress, inflammation, or its cofactor activities.

## Considerations for Future Studies

A number of important issues need to be taken into consideration with respect to design of future studies assessing the efficacy of IV vitamin C on the QOL of cancer patients (Table 2). A major drawback of the QOL studies carried out to date is that none have included a placebo control. Thus, it is not possible to rule out a placebo effect, particularly as this effect tends to be more prevalent with measures of subjective symptoms such as pain (43). Therefore, it is critical that future QOL studies comprise an IV placebo control, either as a parallel arm or as part of a cross-over study design. Although additional phase I–II trials are currently underway, which will assess level of fatigue, pain control, and QOL with IV vitamin C treatment (2, 44), these do not appear to have placebo controls. It is noteworthy that some studies indicate that the dose of vitamin C required for QOL improvement (e.g., 7.5–10 g/session) (6, 7) may be lower than those typically administered by physicians as adjuvant treatment (e.g., 25–100 g/session) (1). The optimum vitamin C dose for QOL improvement has not been determined.

**Table 2:** **Considerations for future quality of life (QOL) studies**.

Considerations	Comments
Placebo control	No QOL studies have used a placebo control
	Ideally, use an IV placebo control as a parallel arm or cross-over study design
IV vitamin C dose	Different IV vitamin C doses have been used to date (e.g. 7.5–100 g/session)
	Ideally, determine minimum dose required as may be significantly lower than doses typically administered as adjuvant treatment (1)
IV vitamin C interval and duration	Different intervals for IV vitamin C administration have been used (e.g. once weekly to daily) and different durations have been utilized (e.g. 1 week to 12 months)
	Ideally, determine duration of effect post-IV vitamin C to inform the number of sessions/week required to obtain a sustained improvement in QOL
IV vitamin C and drug interactions	Some IV vitamin C and chemotherapy combinations have been tested (27, 28, 46)
	Ideally, bracket chemo-/radiotherapy to avoid potential interactions if specific combination has not yet been tested
Patient characteristics	Some studies have combined ± chemo-/radiotherapy (7, 9) and some studies have combined different tumor types (6, 8, 9)
	Ideally, quantify QOL effect with or without adjuvant treatment, and analyze individual tumor types due to varying symptoms
Vitamin C status	No QOL studies have reported patient vitamin C status at baseline or following IV vitamin C administration
	Ideally, measure vitamin C status at screening to stratify participants or exclude from study, or at baseline to facilitate subgroup analysis
QOL measures	Some studies have used EORTC-QLQ-C30 (6, 8, 9) and MFSI (22, 23), while others have used intensity of typical complaints (7)
	Ideally, use standardized measures to facilitate cross study comparisons
Mechanisms	No QOL studies have investigated potential mechanisms of IV vitamin C action
	Ideally, measure appropriate biomarkers of activity (e.g., biomarkers of oxidative stress, inflammation, and cofactor activities)

To date, variable intervals of IV vitamin C administration, from daily to once weekly (typically twice weekly) have been used, and the duration of a course of treatment has also varied greatly, from 1 week to 12 months. None of the QOL studies have determined how long the effect of an individual IV vitamin C session lasts; this would inform the number of sessions required per week to obtain a sustained improvement in QOL. The pharmacokinetics of IV vitamin C has been well characterized in phase I studies of cancer patients (8, 45); plasma vitamin C levels reach a maximum at the end of the infusion, the average duration of which is typically 1.5 h (1), then immediately begin to drop, with a half-life of approximately 2 h (8, 45). Although IV vitamin C administration does not appear to adversely interfere with the chemotherapeutic agents tested to date [gemcitabine, erlotinib, paclitaxel, and carboplatin (27, 28, 46)], it has been suggested that an interval of five half-lives be allowed either side of IV chemotherapy as a strategy for avoiding potential drug interactions (47). Thus, based on its 2 h half-life, infusion of vitamin C on the day prior to chemotherapy would avoid any interactions with chemotherapeutic agents that have not yet been tested in combination with vitamin C.

Other important considerations include patient characteristics, particularly their vitamin C status. Cancer patients exhibit low circulating vitamin C levels (29, 30), despite comparable dietary intakes to healthy controls (48), and also exhibit lower plasma vitamin C status following IV administration (36). This is possibly due to enhanced metabolic turnover of the vitamin due to increased cancer- or chemotherapy-associated oxidative stress and/or inflammation. Although IV vitamin C pharmacokinetics was measured in one of the studies, which assessed QOL, patient status at study entrance or following supplementation was not reported (8). Measurement of patient vitamin C status at screening would allow stratification and also facilitate subgroup analysis or exclusion from the study if vitamin C status is already saturating, i.e., likely abrogating any effect of intervention (49). Some QOL studies have included participants who are undergoing adjuvant treatment as well as those who are not (7, 9). Furthermore, some QOL studies have included patients with cancer at different sites (6, 8, 9). Both of these factors are likely to impact on the type and/or degree of side-effects, which impact on QOL and should be taken into consideration when planning future studies.

## Conclusion

Due to the debilitating nature of cancer- and chemotherapy-related fatigue and other side-effects that impact on QOL, any therapy that can alleviate these symptoms will significantly improve the QOL of sufferers. A number of phase I clinical trials have shown that high-dose IV vitamin is safe and well tolerated in cancer patients, either as a monotherapy (8, 45) or in combination with other chemotherapeutic agents (28, 46). Although direct anti-cancer effects of IV vitamin C have not yet been confirmed by these small studies, there is consistent evidence that IV vitamin C can improve cancer patient’s QOL (6–9) and decrease multiple aspects of fatigue (22, 23). Patients with cancer-related fatigue are often prescribed central nervous system stimulants, despite a limited evidence base, and the fact that these are often ineffective (50). Thus, if future intervention studies indicate that IV vitamin C is an effective anti-fatigue therapy, this will reduce the use of ineffective medications and their accompanying side-effects. Overall, additional well-designed placebo-controlled studies investigating the effects of IV vitamin C on QOL of cancer patients appear warranted.

## Conflict of Interest Statement

The authors declare that the research was conducted in the absence of any commercial or financial relationships that could be construed as a potential conflict of interest.
